# Association of Night-Time Screen-Viewing with Adolescents’ Diet, Sleep, Weight Status, and Adiposity

**DOI:** 10.3390/ijerph19020954

**Published:** 2022-01-15

**Authors:** Chelsea L. Kracht, Jordan Gracie Wilburn, Stephanie T. Broyles, Peter T. Katzmarzyk, Amanda E. Staiano

**Affiliations:** Population and Public Health Science, Pennington Biomedical Research Center, 6400 Perkins Road, Baton Rouge, LA 70808, USA; chelsea.kracht@pbrc.edu (C.L.K.); jorgrawil@gmail.com (J.G.W.); stephanie.broyles@pbrc.edu (S.T.B.); peter.katzmarzyk@pbrc.edu (P.T.K.)

**Keywords:** television, child, obesity, digital media

## Abstract

Night-time screen-viewing (SV) contributes to inadequate sleep and poor diet, and subsequently excess weight. Adolescents may use many devices at night, which can provide additional night-time SV. **Purpose:** To identify night-time SV patterns, and describe differences in diet, sleep, weight status, and adiposity between patterns in a cross-sectional and longitudinal manner. **Methods:** Adolescents (10–16 y) reported devices they viewed at night and completed food recalls. Accelerometry, anthropometrics, and imaging were conducted to measure sleep, weight status, and adiposity, respectively. Latent class analysis was performed to identify night-time SV clusters. Linear regression analysis was used to examine associations between clusters with diet, sleep, weight status, and adiposity. **Results:** Amongst 273 adolescents (12.5 ± 1.9 y, 54% female, 59% White), four clusters were identified: no SV (36%), primarily cellphone (32%), TV and portable devices (TV+PDs, 17%), and multiple PDs (17%). Most differences in sleep and adiposity were attenuated after adjustment for covariates. The TV+PDs cluster had a higher waist circumference than the no SV cluster in cross-sectional analysis. In longitudinal analysis, the primarily cellphone cluster had less change in waist circumference compared to the no SV cluster. **Conclusions:** Directing efforts towards reducing night-time SV, especially TV and PDs, may promote healthy development.

## 1. Introduction

Childhood obesity is a public health concern and affects 20.6% of adolescents (ages 12–19 years) in the United States (US) [1]. Adolescents with obesity are approximately five times more likely to have obesity in adulthood compared to adolescents without obesity [2]. Excess weight in adolescence is also related to a higher likelihood of adult diabetes and coronary heart disease [3]. Screen-time is an important behavior to consider in relation to obesity as a recent study found many U.S. adolescents (ages 13–17 years) have access to a cellphone (95%) or a desktop or laptop computer at home (88%), and about half (45%) report they use the internet constantly [4]. In a nationally representative sample of 24,500 US high school students, excess screen-time was related to an increased likelihood of having obesity, along with unhealthy behaviors including poor diet and inadequate sleep [5]. These findings suggest that screen-time is associated with obesity, likely through negatively impacting diet and sleep. 

Screen-time may influence dietary quality through the excess consumption of nutrient-poor food and excess calories. For example, a cross-sectional study of 659,288 adolescents found excess screen-time was related to higher weight status through nighttime consumption of sugary drinks, fried meats, and various desserts [6]. Another study found the relationship among TV viewing and video-game use with BMI z-score was mediated by higher caloric intake in 283 adolescents (ages 14–16 years) with obesity [7]. Along with its influence on diet, screen-time may also contribute to shorter sleep duration, resulting in excess weight gain. Accordingly, a study amongst 8th and 11th graders found the relationship between screen-time hours and BMI percentile was mediated by short sleep hours in boys but not girls [6]. The relationship between screen-time and short sleep may suggest that it is important to consider the time of day when viewing screens, i.e., viewing screens at night. Indeed, a cross-sectional study of young adolescents (ages 10–11 years) found those who engaged in screen-time at night had a shorter sleep duration and lower diet quality than adolescents who did not view screens at night [8]. 

Different types of screen use may also be an important consideration, as demonstrated in a cross-sectional study of 926 adults, where high television users and high cellphone users had lower dietary quality relative to heavy laptop or tablet users [9]. Further, the number of devices may also need to be considered as multiple device use is becoming common in adolescents [10]. In a cross-sectional study of 643 adolescents, those who viewed multiple devices had less physical activity relative to general computer users and computer gamers. These findings suggest that the number of devices may also differentially impact health behaviors [11]. It is unclear if device type and use patterns, especially those at night, are related to adolescent diet, sleep, and weight status.

In addition to examining cross-sectional associations, systematic reviews have called for longitudinal studies to understand how screen-time impacts diet, sleep, weight status, and adiposity over time [12,13]. Current longitudinal studies examining the relationship between screen-time and development of poor health behaviors, weight, and adiposity are confined to one specific device type (i.e., only TV) [14] and do not include specific use patterns (e.g., at night) [12]. Therefore, this study aimed to: (1) classify adolescent night-time screen-viewing patterns; (2) describe cross-sectional differences in health behaviors (diet and sleep), weight status, and adiposity by night-time screen-viewing patterns; and (3) evaluate the relationship between night-time screen-viewing patterns and changes in health behaviors, weight status, and adiposity in adolescents. 

## 2. Materials and Methods

Adolescents (ages 10–16 years) from a metropolitan area in a southeastern US state were recruited to participate in the Translational Investigation of Growth and Everyday Routines in Kids (TIGER Kids) prospective observational cohort study between 2016–2018, with follow-up measurements occurring approximately 2 years after baseline measures. Recruitment occurred via email listserv, community events, social media, and health fairs. Exclusion criteria for the TIGER Kids study included an adolescent weighing greater than 500 pounds, pregnancy, a medically restrictive diet, and any status that hindered mobility. Retention efforts included phone calls and emails every six months to adolescents and families, and scheduling the follow-up visit a few months in advance. The study was approved by the Pennington Biomedical Research Center’s Institutional Review Board.

Parents provided written informed consent and adolescents provided assent at the baseline visit. At baseline, adolescents were asked to wear an accelerometer for at least seven days and completed up to two dietary recalls prior to the clinic visit. Anthropometry, imaging, and another dietary recall were performed at the clinic also. Parents reported the adolescent’s date of birth (to calculate age), sex, race, household size, and household income (beginning with <$10,000, then in increasing increments of $20,000 until ≥$140,000). Adolescents reported whether they were in or out of school (i.e., summer or holiday) during the measurement period and completed additional questionnaires. All questionnaires were managed using REDCap (Research Electronic Data Capture) hosted at Pennington Biomedical Research Center [15,16]. At the follow-up visit, anthropometric and imaging procedures were completed in the same manner.

Adolescents completed the Screen-Based Media Use Scale at the baseline visit only, which assessed device use and screen-viewing throughout the day [10]. This scale inquired whether the adolescents had viewed the screens during certain times of the day over the last week, including “right after school”, “evening in your free time”, “in bed at night”, and “on weekends”. Responses for “in bed at night” were used for the present analysis. Adolescents were asked whether they used (yes or no options) 11 devices, including, (1) “no screen-viewing”, (2) gaming system (e.g., Xbox, play station, game cube, Wii, Kinect), (3) cellphone (iPhone, Android phone, etc.), (4) TV (watching), (5) TV (used for gaming), (6) Laptop (including notebook, netbook, or Mac), (7) Tablet (iPad, Android, etc.), (8) personal computer (e.g., desktop computer), (9) portable video game device (e.g., PlayStation Portable or Nintendo DS), (10) touch screen music player (e.g., iPod Touch), or (11) eReader (e.g., Kindle, Nook). If the adolescent selected “no screen-viewing” but also selected viewing another screen device (i.e., cellphone), they were classified by the devices they selected rather than “no screen-viewing” (*n* = 6). All night-time screen-viewing options were considered when creating clusters.

Twenty-four-hour dietary recalls of food and beverage intake were used to estimate dietary intake. These recalls were completed using the Automated Self-Administered 24-Hour Dietary Assessment Tool (ASA-24 2016), which is a web-based program created by the National Cancer Institute [17]. Prior to the clinic visit at baseline, the research team emailed the adolescent to complete two separate recalls at preselected intervals (unannounced) so the adolescent could complete one weekday and one weekend recall ahead of the visit. An additional dietary recall was completed at the visit (which happened on a weekday). If one or no dietary recalls were completed before the clinic visit, the adolescent was contacted within 30 days to complete further dietary recalls to obtain their average dietary intake. At follow-up, adolescents were asked to complete up to three dietary recalls prior to the clinic visit, one dietary recall at the clinic visit, and then contacted within 30 days to complete further dietary recalls (maximum six recalls). The ASA-24 utilizes the automated multiple-pass method, which creates multiple prompts within the tool to aid in the recall of food and beverages throughout the day and has been previously validated within this age range [18]. Using ASA-24 output, an SAS macro was used to calculate a score corresponding to the Healthy Eating Index (HEI) 2015 [19]. HEI total score is a calorie-adjusted measurement based upon adherence to the Dietary Guidelines for Americans in 2015. The HEI total score is comprised of 13 subcomponents that are summed to generate a total score of 0 to 100, where a higher score indicates higher diet quality. Average HEI total score and kilocalories from all available days (1–3 possible days at baseline and 1–6 days at follow-up) were used in the analysis.

Accelerometry was used to measure sleep duration. Adolescents were asked to wear an accelerometer on their hip for seven continuous days (24 h/day). A validated algorithm was used to classify sleep and non-wear time in youth [20]. Sleep duration was calculated using the algorithm-derived bedtime and wake time. Overall sleep duration was the average sleep duration from all nights. Weeknight sleep duration was the average of overnight sleep from Monday, Tuesday, Wednesday, and Thursday nights. Adolescents with at least three days (including one weekend day) of 160 sleep minutes were included in the analysis [20]. 

Waist circumference (cm) was measured at a point halfway between the inferior border of the rib cage and the superior aspect of the iliac crest, by a trained researcher to the nearest 0.1 cm. Clothing was moved out of the way for the measurement. A trained researcher also completed height and weight measurements to the nearest 0.1 cm or 0.1 kg, respectively. If the two measurements differed by more than 0.5 units, a third measure was completed. Age- and sex-specific BMI percentiles were calculated based on national reference data [21]. Percent of the 95th percentile was used if adolescents had a BMI percentile ≥98th percentile [22]. Obesity was defined as a BMI percentile ≥95th percentile. A whole-body scan with a General Electric (GE) Lunar iDXA scanner (GE Medical Systems, Milwaukee, WI, USA) using a standard imaging and positioning protocol was used to assess body fat percentage, fat mass, and lean mass. The amount of body fat compared to the total body mass was used to calculate body fat percentage. 

For aim one, latent class analysis was performed to identify clusters of night-time screen-viewing at baseline using the eleven screen-viewing responses. The latent class cluster was defined as the probability of reporting night-time screen-viewing of the specific device. Latent class analysis is a method used to identify latent classes of individuals based on categorical variables [23], which in the present study were defined as “yes” or “no”. Analyses (PROC LCA; SAS v 9.4, Cary, NC, USA) considered 2–7 clusters, and classification metrics were used to determine the best cluster fit (i.e., lower Bayesian information criterion (BIC), Akaike information criterion (AIC), and a higher log-likelihood and entropy (Supplementary Table S1)) [24]. A minimum number of clusters was also considered for the generalizability of results. 

For aim two, a one-way analysis of variance (ANOVA) with a Tukey post-hoc test was used to compare unadjusted differences between clusters in demographics; health behaviors (HEI total score, kilocalories, overall sleep duration, and weeknight sleep duration); weight status (waist circumference and BMI percentile); and adiposity (percent body fat, lean mass, and fat mass). Chi-square or Fisher exact tests for small cell size were used to compare differences between clusters in categorical variables. Multilevel linear regression models, which accounted for the clustering of adolescents within the same household, were used to assess the association between the independent variable of night-time screen-viewing pattern and dependent variables of baseline health behaviors, weight status, and adiposity. Models were adjusted for adolescent age, sex, race, in or out of school status, and household size. Similarly, a multilevel logistic regression model was used to assess the association between the independent variable of night-time screen-viewing cluster and the dependent variable of obesity, with adjustment for the same covariates. 

For aim three, a subsample analysis of those included in aims one and two was conducted. Adolescents with complete measures for health behaviors, weight status, and adiposity at follow-up were included in this analysis. As a total sample, multiple repeated measures ANOVAs were conducted to compare the difference between baseline and follow-up variables, including health behaviors, weight status, and adiposity. As for differences amongst clusters, one-way ANOVA and chi-square tests were used to compare health behaviors, weight status, and adiposity components at both baseline and follow-up between clusters. A Tukey post-hoc test was used for pair-wise comparisons. Multilevel linear regression models, accounting for clustering of adolescents within the same household, were also used to examine the relationship between night-time screen-viewing pattern at baseline with change in health behaviors, weight status, and adiposity components. These models were adjusted for the same covariates as aim two models, along with time between baseline and follow-up (months) and baseline values of health behaviors, weight status, or adiposity. Multilevel logistic regression models were used to examine associations between baseline night-time screen-viewing and obesity status at follow-up with adjustment for the same covariates. There was no difference in health behaviors, weight status, or adiposity at follow-up measurements between measures conducted before the COVID-19 pandemic (March 2020) and during the pandemic (between June and August 2020, *p* > 0.05). A *p*-value of 0.05 was used for significance, and all analyses were performed using SAS 9.4 (Cary, NC, USA).

## 3. Results

In total, 342 participants completed measures, and 273 participants provided complete measures at baseline. Participants were not included in the analysis if they did not complete the night-time screen-viewing questionnaire (*n* = 2), did not complete any dietary recall (*n* = 26), were missing, or did not have adequate wear time for sleep data (*n* = 38), or were missing anthropometric (*n* = 2) or imaging measurements (*n* = 1). Compared to those not included in the analysis, those that were included had a lower BMI percentile (70.4 ± 30.5 vs. 79.8 ± 24.3, *p* = 0.001). There were no other differences in demographics, health behaviors, weight status, or adiposity between those that were included and those that were not included in the analysis (*p* > 0.05 for all).

Overall, adolescents were 12.5 ± 1.9 years of age; 45.8% were male; 59.7% were White and 34.8% were African American; 5.5% identified as another race (e.g., Asian) or identified as two or more races; the majority lived in a household with an income above $70,000 (59.5%); and more than half completed their measurements during the school year (59.2%). On average, adolescents had a low HEI total score (47.8 ± 11.4), reported eating 1761 ± 747 kilocalories/day, and slept 8.7 ± 1.0 h/night overall. Adolescents who reported night-time screen-viewing usually watched more than one screen (1.4 ± 0.8 devices).

### 3.1. Aim 1: Classify Night-Time Screen-Viewing Patterns

**Figure 1:**
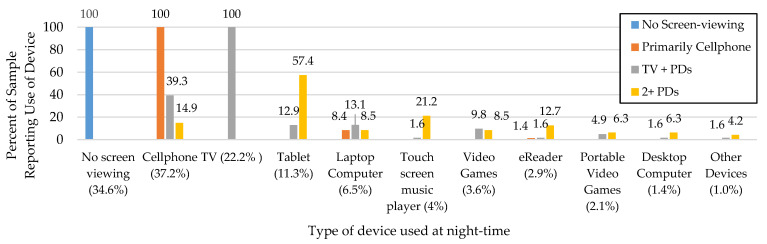
Night-time screen-viewing by cluster and device used Participants could select multiple devices unless they chose no screen-viewing; the proportion shown under each device indicates the amount of total sample using the device at night-time; TV = television; PD = portable device.

The four-cluster model was chosen, considering interpretability and all fit indices to classify night-time screen-viewing patterns (Supplementary Table S1). The four clusters identified included, (1) no screen-viewing, (2) primarily cellphone, (3) TV and portable devices (TV+PDs), and (4) 2 or more PDs with no TV (2+PDs) (Figure 1). Overall, many adolescents reported using a cellphone (37.2%) or TV (22.2%) in bed at night. The no screen-viewing cluster (*n* = 95) did not indicate any screen-viewing and comprised one-third of the sample (34.6%). The second most common cluster was the primary cellphone cluster, and all adolescents in this cluster utilized a cellphone (100%, *n* = 71), with few using a laptop computer (8.4% of cluster) or eReader (1.4% of cluster). All adolescents in the TV+PDs cluster viewed a TV in bed at night (100%, *n* = 60), with some reporting using a cellphone (39.3%) and various use of the eight other portable devices (<15% per device). The smallest cluster was the 2+PDs cluster (17.1% of the sample, *n* = 47), for which the most common device used was a tablet (57.4%, *n* = 27), and these adolescents reported using all other devices except a TV (0%).

### 3.2. Aim 2: Describe the Cross-Sectional Differences in Health Behaviors, Weight Status, and Adiposity by Night-Time Screen-Viewing Patterns

As shown in Table 1, the cross-sectional differences in clusters were in age, race, and weeknight sleep duration (*p* < 0.05 for all). The primarily cellphone cluster was significantly older than all other clusters, and the TV+PDs and 2+PDs clusters were older than the no screen-viewing cluster (*p* < 0.05 for all). No pair-wise differences were found in weeknight sleep duration (*p* > 0.05 for all). As for weight status and adiposity, primarily cellphone and TV+PDs clusters had a higher waist circumference and lean mass compared to the no screen-viewing cluster (*p* < 0.05 for all) at baseline. Further, the TV+PDs cluster had a higher fat mass compared to the no screen-viewing cluster (*p* < 0.05). There were no other differences between clusters (*p* > 0.05 for all). 

After adjustment for demographic characteristics, those in the TV+PDs cluster had a higher waist circumference (*β* = 6.67, *SE* = 3.08, *p* = 0.03) compared to those in the no screen-viewing cluster but no significant difference in adiposity (*p* > 0.05 for all) compared to those in the no screen-viewing cluster. Those in the primarily cellphone cluster did not have a higher waist circumference (*p* = 0.09) or lean mass (*p* = 0.08) compared to the no screen-viewing cluster after adjustment. Age, sex, race, in-and-out of school status, and household size were cross-sectionally related to health behaviors, weight status, and adiposity indicators across the models (Supplementary Table S2).

### 3.3. Aim 3: Association between Night-Time Screen-Viewing Clusters and Changes in Health Behaviors, Weight Status, and Adiposity

Two hundred and seventeen adolescents returned for follow-up measurements (79% of those included in baseline analysis). Participants who did not return were unable to be contacted or located (*n* = 41), moved out of area (*n* = 6), had a school or time conflict (*n* = 4), withdrew from the study (*n* = 1), refused to give reason (*n* = 2), or changed their mind (*n* = 1). Within those who did return (*n* = 217), those with incomplete accelerometry data (*n* = 54), dietary recalls (*n* = 11), or imaging procedures (*n* = 1) were not included in analysis. Those who were included in analysis had lower fat mass (*n* = 151, 42.85 ± 10.18 kg) compared to those who had incomplete data (*n* = 65, 46.38 ± 11.07 kg, *p* = 0.01). There were no other differences in demographic characteristics, health behaviors, weight status, or adiposity between those included and not included in follow-up analysis (*p* > 0.05 for all). Clusters differed in age, race, waist circumference, lean mass, and fat mass at baseline within the longitudinal sample (*p* < 0.05 for all, Supplementary Table S3).

**Figure 2:**
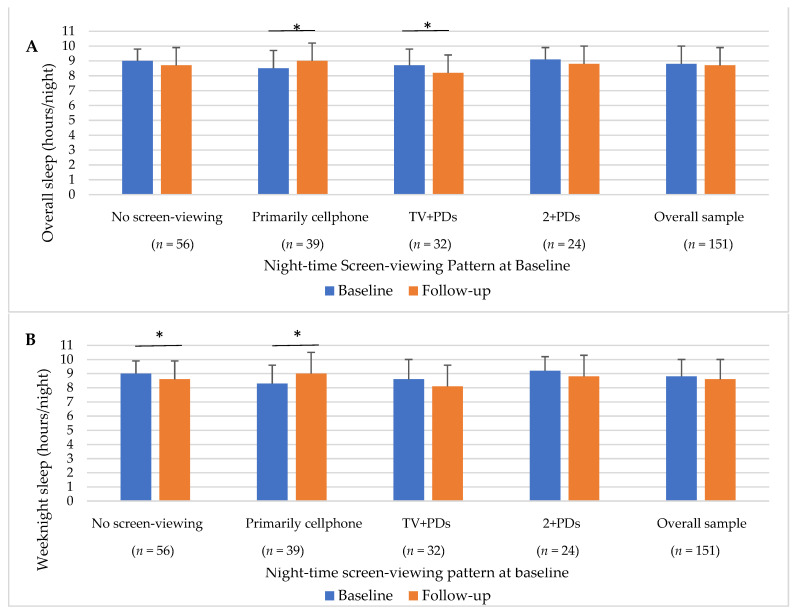
Changes in overall and weeknight sleep by night-time screen-viewing pattern. (**A**) *Overall Sleep*; (**B**) *Weeknight Sleep*; values presented are unadjusted means and standard deviations; * significant difference between baseline and follow-up using paired t-test within group.

In unadjusted models, there was a significant effect of time and cluster on overall sleep and weeknight sleep (Figure 2). In the longitudinal sample (*n* = 151), there was no significant increase in overall or weeknight sleep (*p* > 0.05 for both). When considering within clusters, the no screen-viewing cluster decreased their weeknight sleep between baseline and follow-up (−0.41 ± 1.40 h/night, *p* = 0.03), while the primarily cellphone cluster increased their overall (0.53 ± 1.59 h/night, *p* = 0.04) and weeknight sleep (0.72 ± 1.85 h/night, *p* = 0.02). The TV+PDs cluster’s overall sleep decreased between baseline and follow-up (−0.55 ± 1.30 h/night, *p* = 0.02). As for diet, the no screen-viewing cluster decreased their HEI total score (−5.15 ± 11.75, *p* = 0.001) between baseline and follow-up. There were no other differences in health behaviors within or between clusters from baseline and follow-up.

As for weight status and adiposity, there was a significant increase in waist circumference (5.55 ± 11.00, *p* = 0.001), BMI percentile (4.20 ± 16.8, *p* = 0.002), lean mass (6.59 ± 5.25, *p* = 0.001), and fat mass (3.66 ± 6.30, *p* = 0.001) but no difference in body fat percent (−0.20 ± 5.40, *p* = 0.63) in the full sample. Within clusters, the no screen-viewing, TV+PDs, and 2+PDs clusters reported a significant increase in waist circumference (*p* < 0.05 for all). Further, each cluster had an increase in lean and fat mass (*p* < 0.05 for all). 

In adjusted models to assess the association between night-time screen-viewing and health behaviors, weight status, and adiposity, those in the primarily cellphone cluster had less change in their waist circumference (*β* = −5.56, *SE* = 0.80, *p* = 0.04) compared to the no screen-viewing cluster. Though non-significant (*p* > 0.05 for all), those in the TV+PDs cluster decreased their overall sleep (*β* = −0.51, *SE* = 0.27, *p* = 0.06) and those in the primarily cellphone cluster increased their weeknight sleep (*β* = 0.63, *SE* = 0.35, *p* = 0.08) compared to those in the no-screen-viewing cluster. Further, the TV+PDs cluster increased their fat mass (*β* = 2.92, *SE* = 1.49, *p* = 0.05) relative to the no screen-viewing cluster. No other significant associations were found between night-time screen-viewing cluster, health behaviors, weight status, and adiposity at baseline or follow-up after adjusting for covariates in the longitudinal sample.

## 4. Discussion

The purpose of this study was to identify night-time screen-viewing patterns in adolescents and describe differences amongst these patterns in a cross-sectional and longitudinal manner. This study identified four distinct patterns of night-time screen-viewing in bed amongst a diverse cohort of adolescents. Some adolescents had no night-time screen-viewing, but most used multiple devices, including cellphones and portable devices. In cross-sectional analysis, clusters differed by age, race, weeknight sleep, waist circumference, and adiposity; accounting for other covariates such as age and race attenuated most differences between clusters. In the longitudinal analysis, clusters reported changes in sleep, weight status, and adiposity, but only differences in weight status remained after adjustment. Still, those who viewed fewer screens (i.e., no screens or primarily cellphone) saw beneficial changes to their future physical development.

Specific to identifying night-time screen-viewing clusters, many adolescents in the current sample viewed screens in bed at night, with only one-third having no night-time screen-viewing. These results may reflect the younger adolescent age, as the no screen-viewing cluster was younger than the other clusters, and parental restrictions on night-time screen-viewing may be stricter in younger adolescents [25]. It may be expected that cellphone and TV would be prevalent with the portability of the cellphone and the option of a TV in the bedroom. A report in older adolescents (ages 13–17 years) found many used at least one screen at night, namely cellphones (86% had access and used) [26]. This amount is noticeably more than the current sample (37.2%), which may be due to the younger age range (10–16 years) in the current sample. The third most common device was a tablet, which combines the properties of both a TV and cellphones. Tablets are portable with larger screens and device storage but may be subject to parental controls for use, including access in the bedroom or permissions on the tablet limiting their use during bed-time hours [27]. One possible explanation for the range of device use may be the timing of the academic year. A cross-sectional study of 146 adolescents (16.4 ± 1.0 years) found night-time screen-viewing use differs by in school and out of school status (i.e., summer), with more cellphone use during the in school period and more video games or TV during the out of school period [28]. The current sample was roughly equally divided amongst in and out of school periods, suggesting other contextual factors may warrant consideration. 

Considering the variety of devices viewed while in bed, the identified clusters within the current sample suggest the purpose of night-time screen-viewing may differ amongst adolescents. Some adolescents only used cellphones, while others used this device in tandem with multiple devices. One potential reason is the use of devices for communication and socialization compared to entertainment. TVs and video game consoles may have internet connections for some social opportunities, but these devices are primarily used to watch and engage in extended entertainment periods (i.e., TV shows). However, cellphones serve a primary purpose for communication and can be used for socialization in both short and long periods. The different features of these devices may also clarify the screen-viewing patterns identified, as found in the cross-sectional differences between clusters: the primarily cellphone cluster was older than other clusters and included slightly more girls. As detailed in a cross-sectional study of 362 adolescents (12–17 years), more girls used media at night for calling and texting in bed before sleep, and fewer girls used TV before bedtime compared to boys [29]. Therefore, the weight status differences in the identified clusters may be representative of both the social intent and demographic characteristics of adolescents.

The demographic characteristics amongst the cluster and device properties are important factors when considering individual cluster results, especially the longitudinal results. The primary cellphone cluster reported increases in sleep from baseline to follow-up and less change in weight status compared to the no screen-viewing cluster. These results may be due to age, which did differ amongst clusters at baseline. The primarily cellphone cluster was the oldest cluster and may be more stable in their sleep trajectory compared to other clusters (e.g., no cellphone cluster). Not considering age, the changes in sleep amongst the primarily cellphone cluster is unexpected as cellphones may contribute to night awakenings, thus, shortened sleep. Amongst 846 adolescents, those with night-awakenings from their cellphones at baseline were three times more likely to have problems falling asleep and restless sleep one year later [30]. However, cellphones’ have advanced at a rapid rate and there are now settings (e.g., sleep mode) to prevent these awakenings and therefore less disturbance to sleep duration. Another finding was that the primarily cellphone cluster saw less of a change in waist circumference compared to those who did not view screens over two years. This difference indicates the primarily cellphone cluster maintaining their waist circumference, which may occur at an older age and not necessarily that they lost weight over this period. We caution interpretation that primarily cellphones have healthier patterns, as no significant differences were found in health behaviors between these clusters over two years when adjusting for covariates.

However, others have found that primarily cellphone users are like those who do not view screens. Amongst Brazilian adolescents (*n* = 574), high cellphone users were not different than low screen-time users in their waist circumference, whereas other screen users (gaming and high screen-time) reported a higher waist circumference compared to low screen-time users [31]. The use of the cellphone for intermittent communication may mean less time is spent on this device compared to a TV or video game console, though the duration of time spent was not captured in the present study and warrants further investigation. Like the current study, differences in weight status and adiposity in that study were attenuated when accounting for demographic characteristics [32]. Aside from age, another important demographic characteristic when considering adolescent weight status and adiposity is race. Differences in waist circumference between the TV+PDs cluster and the no screen-viewing cluster may be indicative of a higher number of African American adolescents in the TV+PDs cluster, rather than a biological mechanism. A nationally representative study of children (ages 6–17 years) found African American adolescents were more likely to have a TV in the bedroom compared to others [33], along with another study of 369 children and adolescents that found those with a TV in the bedroom were at risk for a high waist circumference [34]. 

Though differences were found in sleep between clusters, in both cross-sectional and longitudinal results, there were no differences in diet or between the 2+PDs cluster and other clusters in health behaviors, weight status, or adiposity. This lack of difference in dietary outcomes suggests these other factors (e.g., age, sex, household quantity) may better characterize health behaviors and weight status more than their night-time screen-viewing patterns. For example, night-time screen-viewing may not directly impact diet, but older children may have a less healthy diet and view more screens in bed compared to younger children. Still, the difference amongst night-time screen-viewing clusters, namely those who viewed multiple screens, in waist circumference, may propose continued unhealthy dietary behaviors and energy imbalance [32]. The 2+PDs cluster used various devices, including tablets, touch screen music players, and cellphones, thus, may incorporate demographic and social interests of both the primarily cellphone and TV+PDs clusters. Nonetheless, those who viewed fewer screens (primarily cellphone and no screen-viewing) reported healthier behaviors, weight status, and adiposity relative to those who viewed multiple devices (i.e., TV+PDs and 2+PDs cluster).

Strengths of the current study include a large and diverse sample (34.8% African American, 5.5% other race), which is a comparable proportion to a nationally representative cohort of US adolescents (ages 12–19 years, 34.4% African American, 4.4% other race) [12], the use of device-based measures for sleep, imaging methods to assess adiposity, and a longitudinal study design. Device-based measures of sleep duration may provide a more accurate measure of actual sleep relative to parent-reported sleep [35], thus, the current study expands upon previous screen-viewing research using parent and self-reported sleep [5,6,8]. The current study also assessed multiple common devices (e.g., eReader, cellphone, PlayStation), rather than only one device (e.g., TV) to address deficits highlighted in other screen-time research that solely focused on TV [5]. Still, one limitation of the current study is that additional information on the length of device usage and concurrent usage at night was not assessed but could provide additional context, as well as the purpose of screen-viewing and potential exposure to food advertisements or other content that may influence adiposity and health behaviors [10]. These reports are also historical and diary-based, and a limitation is that there was no objective assessment of the use of these devices at night. A consideration and another limitation for the current study is the potential underreporting of energy intake as adolescents with higher BMI percentiles may underreport energy intake [36], and much of the sample had overweight (14.2%) or obesity (34.27%). However, the current ASA-24 2016 utilizes an updated automated multiple-pass method [17] since the publication of Singh (2009) [36], which may increase the reporting of food items through prompting multiple recalls. The current study also used HEI total score, which is a calorie-adjusted score of diet quality. Though this study obtained longitudinal measures, a limitation is there was no assessment of night-time screen-viewing at follow-up. Therefore, it is unclear if patterns changed between baseline and follow-up. Further, long-term short sleep or sleep deprivation may contribute to obesity, but the current study was limited to measures at two timepoints almost 2-years apart. Other psychological considerations such as addiction to screen-time devices were also not captured in the current study. Another limitation is only 55% (*n* = 151) of the original sample in analysis (*n* = 273) had complete data at follow-up, and smaller groups were used for longitudinal analysis (range: 24–56 people per cluster). The changes over time were small and may be subject to chance and other maturation processes not measured in the current sample as well.

These findings suggest three specific next steps in research to improve measurement, assessment of sleep lost, and changing screen-time habits. First, future studies could use ecological momentary assessment (EMA), which is a tool that assesses health behaviors in real-world environments using electronic surveys and device-based measures [37]. Though the TIGER Kids study did collect EMA measures during baseline measurements, these EMA methods were specific to daytime activities and did not occur beyond 8:00 p.m. to not interrupt the adolescent’s bedtime routine or sleep [38]. EMA methods may improve the validity and reliability of dietary assessment by assessing diet through event-based and time-based sampling [39], especially in individuals with overweight or obesity [40]. Second, opportunities to identify the amount of sleep delayed, level of sleep deprivation, or lost from viewing screens at night may help quantify the impact of screen-time on adolescent sleep. Recent use of isotemporal substitution modeling, whereby substituting one behavior for another such as sleep and screen-time, is another statistical technique that can assess the relationship between screen-viewing, sleep, and excess weight [41]. Adoption of this technique within the adolescent population could identify the impact of sleep, screen-time, and overall sedentary behavior in the adolescent population. Finally, continued modern assessment of screen-time is needed within a larger and diverse longitudinal cohort. Modern assessment should include measurement of psychological variables, such as problematic use and addiction, and length of use, such as binge-watching. Examination of multiple time points, context, duration, and asking about usage of the latest types of devices may help better understand adolescents’ use of screens in bed at night and the relationship between night screen-time and adolescent health behaviors, weight status, and adiposity. 

## 5. Conclusions

Overall, this study found that one-third of the adolescents viewed screens at night in bed, which may include multiple devices. This study contributes to the evidence that night-time screen-viewing is nuanced, with differing associations between health behaviors, weight status, and adiposity when viewed at one time point and after 2 years. Health professionals should encourage reducing or eliminating night-time screen-viewing and promoting a healthy diet and sleep hygiene for appropriate adolescent growth. 

## Figures and Tables

**Table 1:** Descriptive characteristics of the sample by night-time screen-viewing cluster (*n* = 273) ^.

	No Screen Viewing (*n* = 95)		Primarily Cellphone (*n* = 71)		TV + PDs (*n* = 60)		2+ PDs (*n* = 47)		
	Mean ± SD	%	Mean ± SD	%	Mean ± SD	%	Mean ± SD	%	*p*-value
Age	11.7 ± 1.6		13.5 ± 1.9		12.6 ± 2.1		12.6 ± 1.7		<0.0001 *
Male		51.6		38.0		41.7		51.1	0.26
Race									<0.0001 *
White		64.2		66.2		35.0		72.3	
African American		32.6		28.2		61.7		14.9	
Other		3.2		5.6		3.3		12.8	
Household income									0.60
<$29,999		6.3		9.9		13.2		8.5	
$30,000-$69,999		25.3		19.7		34.0		31.9	
$70,000-$139,000		37.8		40.8		32.0		27.7	
$140,000 or more		23.2		28.2		20.8		29.8	
No response		7.4		1.4		11.5		2.1	
Household size	4.7 ± 1.4		4.1 ± 1.4		4.3 ± 1.5		4.3 ± 1.2		0.09
In-school		56.8		63.4		56.6		59.6	0.85
Devices viewed at night	0.0 ± 0.0		1.1 ± 0.3		1.8 ± 0.8		1.4 ± 1.0		<0.0001 *
*Health Behaviors*									
HEI total score	49.9 ± 11.4		47.2 ± 12.2		46.1 ± 10.1		46.5 ± 11.8		0.16
Kilocalories	1751.6 ± 668.1		1795.8 ± 922.8		1706.9 ± 731.1		1802.9 ± 636.5		0.90
Overall sleep duration (hours)	8.8 ± 0.9		8.7 ± 1.2		8.5 ± 1.2		8.9 ± 0.9		0.14
Weeknight sleep duration (hours)	8.9±1.0		8.5±1.5		8.4±1.4		8.9±1.1		0.03 *
*Weight Status*									
Waist circumference (cm)	74.0 ± 16.2		81.7 ± 18.3		83.6 ± 22.0		77.9 ± 15.8		0.004 *
BMI percentile	73.4 ± 39.1		82.0 ± 38.9		87.4 ± 53.6		77.5 ± 40.7		0.23
BMI category									0.54
Underweight		3.1		1.4		5.0		2.1	
Normal		53.6		49.3		38.3		51.0	
Overweight		15.8		15.5		11.7		12.7	
Obesity		27.3		33.8		45.0		34.0	
Body fat (%)	33.2 ± 9.7		34.7 ± 10.9		36.0 ± 11.9		33.9 ± 10.8		0.45
Lean mass (kg)	33.4 ± 9.6		40.9 ± 10.3		38.9 ± 10.6		36.9 ± 8.6		<0.001 *
Fat mass (kg)	18.5 ± 12.2		23.9 ± 14.6		25.6 ± 14.5		21.0 ± 12.0		0.01 *

^ Assessed using a One-Way analysis of variance (ANOVA) for continuous variables and a chi-squared or Fisher’s exact test for categorical variables; TV = television; PD = portable device; HEI = healthy eating index; BMI = body mass index; * *p* < 0.05.

## Data Availability

Data will be provided upon request.

## Supplementary Materials

The following supporting information can be downloaded at: https://www.mdpi.com/article/10.3390/ijerph19020954/s1, Table S1: Model fit characteristics of clusters identified using latent class analysis, Table S2: Association between of night-time screen-viewing patterns, health behaviors, weight status, and adiposity (*n* = 274), Table S3: Descriptive characteristics of the sample by night-time screen-viewing cluster included in the longitudinal analysis (*n* = 151).

## References

[1] Hales C., Carroll M.D., Fryar C.D., Ogden C.L. (2017). Prevalence of Obesity among Adults and Youth: United States, 2015–2016. NCHS Data Brief.

[2] Simmonds M., Llewellyn A., Owen C., Woolacott N. (2015). Predicting Adult Obesity from Childhood Obesity: A Systematic Review and Meta-Analysis. Obes. Rev..

[3] Llewellyn A., Simmonds M.C., Owen C., Woolacott N. (2016). Childhood Obesity as a Predictor of Morbidity in Adulthood: A Systematic Review and Meta-Analysis. Obes. Rev..

[4] Anderson M.J., Jingjing J. (2018). Teens, Social Media, & Technology 2018. https://www.pewresearch.org/internet/2018/05/31/teens-social-media-technology-2018/.

[5] Kenney E.L., Gortmaker S.L. (2017). United States Adolescents’ Television, Computer, Videogame, Smartphone, and Tablet Use: Associations with Sugary Drinks, Sleep, Physical Activity, and Obesity. J. Pediatr..

[6] Cha E.M., Hoelscher D.M., Ranjit N., Chen B., Gabriel K.P., Kelder S., Saxton D.L. (2018). Effect of Media Use on Adolescent Body Weight. Prev. Chronic Dis..

[7] Cameron J.D., Maras D., Sigal R.J., Kenny G.P., Borghese M.M., Chaput J.-P., Alberga A.S., Goldfield G.S. (2016). The Mediating Role of Energy Intake on the Relationship between Screen Time Behaviour and Body Mass Index in Adolescents with Obesity: The Hearty Study. Appetite.

[8] Chahal H., Fung C., Kuhle S., Veugelers P.J. (2013). Availability and Night-Time Use of Electronic Entertainment and Communication Devices are Associated with Short Sleep Duration and Obesity among Canadian Children. Pediatr. Obes..

[9] Vizcaino M., Buman M., DesRoches T., Wharton C. (2020). From TVs to Tablets: The Relation between Device-Specific Screen Time and Health-Related Behaviors and Characteristics. BMC Public Health.

[10] Houghton S., Hunter S.C., Rosenberg M., Wood L., Zadow C., Martin K., Shilton T. (2015). Virtually Impossible: Limiting Australian Children and Adolescents Daily Screen Based Media Use. BMC Public Health.

[11] Straker L., Smith A., Hands B., Olds T., Abbott R. (2013). Screen-Based Media Use Clusters are Related to Other Activity Behaviours and Health Indicators in Adolescents. BMC Public Health.

[12] Fletcher E.A., Carson V., McNaughton S., Dunstan D.W., Healy G., Salmon J. (2017). Does Diet Mediate Associations of Volume and Bouts of Sedentary Time with Cardiometabolic Health Indicators in Adolescents?. Obesity.

[13] Kracht C.L., Joseph E.D., Staiano A.E. (2020). Video Games, Obesity, and Children. Curr. Obes. Rep..

[14] Falbe J., Rosner B., Willett W.C., Sonneville K.R., Hu F.B., Field A.E. (2013). Adiposity and Different Types of Screen Time. Pediatrics.

[15] Harris P.A., Taylor R., Thielke R., Payne J., Gonzalez N., Conde J.G. (2009). Research Electronic Data Capture (REDCap)—A Metadata-Driven Methodology and Workflow Process for Providing Translational Research Informatics Support. J. Biomed. Inform..

[16] Harris P.A., Taylor R., Minor B.L., Elliott V., Fernandez M., O’Neal L., McLeod L., Delacqua G., Delacqua F., Kirby J. (2019). The REDCap Consortium: Building an International Community of Software Platform Partners. J. Biomed. Inform..

[17] Subar A.F., Kirkpatrick S.I., Mittl B., Zimmerman T.P., Thompson F.E., Bingley C., Willis G., Islam N.G., Baranowski T., McNutt S. (2012). The Automated Self-Administered 24-Hour Dietary Recall (ASA24): A Resource for Researchers, Clinicians, and Educators from the National Cancer Institute. J. Acad. Nutr. Diet..

[18] Moshfegh A.J., Rhodes D.G., Baer D.J., Murayi T., Clemens J.C., Rumpler W.V., Paul D.R., Sebastian R., Kuczynski K.J., Ingwersen L.A. (2008). The US Department of Agriculture Automated Multiple-Pass Method Reduces Bias in the Collection of Energy Intakes. Am. J. Clin. Nutr..

[19] Panizza C.E., Shvetsov Y.B., Harmon B.E., Wilkens L.R., Le Marchand L., Haiman C., Reedy J., Boushey C.J. (2018). Testing the Predictive Validity of the Healthy Eating Index-2015 in the Multiethnic Cohort: Is the Score Associated with a Reduced Risk of All-Cause and Cause-Specific Mortality?. Nutrients.

[20] Tudor-Locke C., Mire E.F., Barreira T.V., Schuna J.M., Chaput J.-P., Fogelholm M., Hu G., Kurpad A., Kuriyan R., for the ISCOLE Research Group (2015). Nocturnal Sleep-Related Variables from 24-h Free-Living Waist-Worn Accelerometry: International Study of Childhood Obesity, Lifestyle and the Environment. Int. J. Obes. Suppl..

[21] Kuczmarski R.J., Ogden C.L., Guo S.S., Grummer-Strawn L.M., Flegal K.M., Mei Z., Wei R., Curtin L.R., Roche A.F., Johnson C.L. (2002). 2000 CDC Growth Charts for the United States: Methods and Development. Vital-Health. Stat. Ser. 11 Data Natl. Health Surv..

[22] Flegal K.M., Wei R., Ogden C.L., Freedman D.S., Johnson C.L., Curtin L.R. (2009). Characterizing Extreme Values of Body Mass Index–for-Age by Using the 2000 Centers for Disease Control and Prevention Growth Charts. Am. J. Clin. Nutr..

[23] Lanza S.T., Collins L., Lemmon D.R., Schafer J.L. (2007). PROC LCA: A SAS Procedure for Latent Class Analysis. Struct. Equ. Model. Multidiscip. J..

[24] Kongsted A., Nielsen A.M. (2017). Latent Class Analysis in Health Research. J. Physiother..

[25] Arundell L., Parker K., Timperio A., Salmon J., Veitch J. (2020). Home-Based Screen Time Behaviors amongst Youth and Their Parents: Familial Typologies and Their Modifiable Correlates. BMC Public Health.

[26] Smith C., de Wilde T., Taylor R.W., Galland B.C. (2020). Prebedtime Screen Use in Adolescents: A Survey of Habits, Barriers, and Perceived Acceptability of Potential Interventions. J. Adolesc. Health.

[27] Toh S.H., Howie E.K., Coenen P., Straker L.M. (2019). “From the Moment I Wake Up I will Use It… Every Day, Every Hour”: A Qualitative Study on the Patterns of Adolescents’ Mobile Touch Screen Device use From Adolescent and Parent Perspectives. BMC Pediatr..

[28] Harbard E., Allen N.B., Trinder J., Bei B. (2016). What’s Keeping Teenagers Up? Prebedtime Behaviors and Actigraphy-Assessed Sleep Over School and Vacation. J. Adolesc. Health.

[29] Lemola S., Perkinson-Gloor N., Brand S., Dewald-Kaufmann J.F., Grob A. (2015). Adolescents’ Electronic Media Use at Night, Sleep Disturbance, and Depressive Symptoms in the Smartphone Age. J. Youth Adolesc..

[30] Foerster M., Henneke A., Chetty-Mhlanga S., Röösli M. (2019). Impact of Adolescents’ Screen Time and Nocturnal Mobile Phone-Related Awakenings on Sleep and General Health Symptoms: A Prospective Cohort Study. Int. J. Environ. Res. Public Health.

[31] da Costa B.G., Salmon J., Dos Santos P.C., Minatto G., Silva K.S. (2020). Clustering of Screen Time Behaviours in Adolescents and Its Association with Waist Circumference and Cardiorespiratory Fitness. J. Sci. Med. Sport.

[32] Gingras V., Rifas-Shiman S.L., Taveras E.M., Oken E., Hivert M.-F. (2018). Dietary Behaviors throughout Childhood are Associated with Adiposity and Estimated Insulin Resistance in Early Adolescence: A Longitudinal Study. Int. J. Behav. Nutr. Phys. Act..

[33] Sisson S.B., Broyles S.T., Newton R.L., Baker B.L., Chernausek S.D. (2011). TVs in the Bedrooms of Children: Does It Impact Health and Behavior?. Prev. Med..

[34] Staiano A., Harrington D.M., Broyles S.T., Gupta A., Katzmarzyk P.T. (2013). Television, Adiposity, and Cardiometabolic Risk in Children and Adolescents. Am. J. Prev. Med..

[35] Gozal D., Dayyat E.A., Spruyt K., Molfese D.L. (2011). Sleep Estimates in Children: Parental Versus Actigraphic Assessments. Nat. Sci. Sleep.

[36] Singh R., Martin B.R., Hickey Y., Teegarden R., Campbell W.W., Craig B.A., Schoeller D.A., Kerr D.A., Weaver C.M. (2009). Comparison of Self-Reported and Measured Metabolizable Energy Intake with Total Energy Expenditure in Overweight Teens. Am. J. Clin. Nutr..

[37] Shiffman S., Stone A.A., Hufford M.R. (2008). Ecological Momentary Assessment. Annu. Rev. Clin. Psychol..

[38] Kracht C.L., Beyl R.A., Maher J.P., Katzmarzyk P.T., Staiano A.E. (2021). Adolescents’ Sedentary Time, Affect, and Contextual Factors: An Ecological Momentary Assessment Study. Int. J. Behav. Nutr. Phys. Act..

[39] Maugeri A., Barchitta M. (2019). A Systematic Review of Ecological Momentary Assessment of Diet: Implications and Perspectives for Nutritional Epidemiology. Nutrients.

[40] Chmurzynska A., Młodzik-Czyżewska M., Malinowska A., Czarnocinska J., Wiebe D.J. (2018). Use of a Smartphone Application Can Improve Assessment of High-Fat Food Consumption in Overweight Individuals. Nutrients.

[41] Stamatakis E., Rogers K., Ding D., Berrigan D., Chau J., Hamer M., Bauman A. (2015). All-Cause Mortality Effects of Replacing Sedentary Time with Physical Activity and Sleeping Using an Isotemporal Substitution Model: A Prospective Study of 201,129 Mid-Aged and Older Adults. Int. J. Behav. Nutr. Phys. Act..

