# Supplementary material for: Association of Night-Time Screen-Viewing with Adolescents’ Diet, Sleep, Weight Status, and Adiposity
Source: Int J Environ Res Public Health. 2022 Jan 15;19(2):954. doi: 10.3390/ijerph19020954 (PMC8775933; doi:10.3390/ijerph19020954)
Supplement: Supplementary file 1 [file ijerph-19-00954-s001.zip › ijerph-1525464-supplementary.pdf]

Supplementary Table S1. Model Fit Characteristics of Clusters Identified using Latent Class Analysis

Number of Clusters Identified	Log Likelihood	AIC	BIC	CAIC	Adjusted BIC	Entropy	Degrees of freedom	Cluster Sizes (range)
2	-729.13	287.73	370.83	393.83	297.90	1.00	2024	96-177
3	-697.95	249.37	375.83	410.83	264.85	0.90	2012	87-96
4	-678.49	234.45	404.27	451.27	255.24	0.92	2000	45-96
5	-664.98	231.43	444.61	503.61	257.53	0.96	1988	8-96
6	-652.42	230.31	486.85	557.85	261.72	0.97	1976	8-96
7	-652.23	253.94	553.83	636.83	290.65	0.97	1964	8-96

AIC = Akaike information criterion; BIC = Bayesian information criterion; CAIC = Consistent Akaike information criterion

Supplementary Table S2. Association between of Night-time Screen-viewing patterns, health behaviors, weight status, and adiposity (*n*=274)

	Overall Sleep Duration			Weeknight Sleep Duration		
	Beta	*95% CI*	*p*-value	Beta	*95% CI*	*p*-value
Cluster						
No screen-viewing	Ref	Ref	Ref	Ref	Ref	Ref
Primarily Cellphone	-0.13	-0.46, 0.20	0.43	-0.14	-0.54, 0.24	0.45
TV and PDs	-0.10	-0.43, 0.24	0.56	-0.15	-0.56, 0.24	0.43
2+ PDs	0.04	-0.31, 0.40	0.81	0.10	-0.32, 0.52	0.64
Age	-0.04	-0.11, 0.02	0.20	-0.11	-0.19, -0.03	0.005*
Sex	-0.02	-0.27, 0.22	0.81	-0.19	-0.49, 0.09	0.19
Race						
White	Ref	Ref	Ref	Ref	Ref	Ref
African American	-0.62	-0.89, -0.35	0.001*	-0.63	-0.95, -0.31	0.001*
Other	-0.16	-0.71, 0.38	0.55	-0.02	-0.67, 0.62	0.93
In-school Status	-0.32	-0.57, -0.07	0.01*	-0.62	-0.92, -0.33	0.001*
Household Size	-0.06	-0.15, 0.02	0.15	-0.04	-0.15, 0.05	0.39
	HEI Score			Kilocalories		
Cluster						
No screen-viewing	Ref	Ref	Ref	Ref	Ref	Ref
Primarily Cellphone	-1.97	-5.78, 1.83	0.30	55.05	-188.20, 298.30	0.65
TV and PDs	-3.38	-7.24, 0.46	0.08	-14.00	-260.46, 232.45	0.91
2+ PDs	-2.95	-7.07, 1.15	0.15	12.80	-249.86, 275.48	0.92
Age	-0.52	-1.29, 0.24	0.17	1.89	-47.04, 50.83	0.93
Sex	1.09	-1.73, 3.92	0.44	-303.29	-483.84, -122.74	0.001*
Race						
White	Ref	Ref	Ref	Ref	Ref	Ref
African American	0.22	-2.82, 3.31	0.88	-87.64	-284.52, 109.24	0.38
Other	1.35	-4.86, 7.57	0.66	72.71	-324.35, 469.79	0.36
In-school Status	2.48	-0.34, 5.32	0.08	-210.07	-391.00, -291.12	0.02
Household Size	0.19	-0.81, 1.20	0.70	-66.78	-131.26, -2.29	0.04*
	BMI Percentile			Body Fat (%)		
Cluster						
No screen-viewing	Ref	Ref	Ref	Ref	Ref	Ref
Primarily Cellphone	9.13	-4.76, 23.03	0.19	2.19	-1.16, 5.54	0.20
TV and PDs	8.43	-5.69, 22.56	0.24	2.51	-0.89, 5.93	0.14
2+ PDs	7.81	-7.19, 22.81	0.30	1.79	-1.82, 5.42	0.33
Age	-1.86	-4.68, 0.91	0.18	-1.07	-1.75, -0.40	0.001*
Sex	10.92	0.60, 21.25	0.03*	6.34	3.84, 8.83	0.001*
Race						
White	Ref	Ref	Ref	Ref	Ref	Ref
African American	17.36	6.10, 28.62	0.002*	1.39	-1.32, 4.11	0.31
Other	-2.41	-25.10, 20.27	0.83	-1.71	-7.19, 3.76	0.53
In-school Status	8.95	-1.38, 19.29	0.09	2.52	0.02, 5.02	0.04*
Household Size	-3.09	-6.78, 0.58	0.09	-0.62	-1.51, 0.26	0.16

*^Assessed using linear regression with additional adjustment for clustering of adolescents within the same household; TV=television; PD=portable device; p<0.05**

Supplementary Table S3. Descriptive characteristics of sample by Night-time Screen-viewing Cluster included in longitudinal analysis (*n*=151) ^

	No screen viewing (*n*=56)		Primarily Cellphone (*n*=39)		TV + PDs (*n*=32)		2+ PDs (*n*=24)		
Baseline Values	Mean ± SD	%	Mean ± SD	%	Mean ± SD	%	Mean ± SD	%	*p*-value
Age	11.4±1.5		14.0±1.7		12.2±1.9		12.0±1.4		<.0001*
Male		55.4		28.2		37.5		41.7	0.06
Race									<.0001*
White		69.6		69.2		31.3		62.5	
African American		25.0		25.6		62.5		16.7	
Other		5.4		5.1		6.25		20.8	
Household Income									0.13
<$29,999		3.6		12.8		15.6		8.3	
$30,000-$69,999		19.6		15.4		31.2		37.5	
$70,000-$139,000		46.4		30.8		18.9		25.0	
$140,000 or more		23.2		38.4		25.0		25.0	
No response		7.2		2.6		9.3		4.2	
Household Size	4.5±1.3		4.2±1.4		4.0±1.3		4.1±1.2		0.23
In-school		42.9		35.9		34.4		37.5	0.86
Devices viewed at night	0.0±0.0		1.1±0.3		1.8±0.8		1.5±1.3		<.0001*
Diet, weight status, and adiposity at baseline									
HEI total score	51.1±11.3		46.0±10.7		46.3±9.3		47.1±12.4		0.09
Kilocalories	1844.7±555.8		1807.4±742.2		1667.0±809.9		1778.7±534.2		0.68
Waist circumference (cm)	70.8±14.1		82.4±19.8		80.8±21.1		78.7±17.9		0.001*
BMI Percentile	65.8±36.3		79.9±40.2		82.9±51.2		80.6±41.3		0.18
BMI category									0.58
Underweight		3.6		0.0		6.3		4.2	
Normal		60.7		53.9		40.6		50.0	
Overweight		10.7		15.3		9.4		8.3	
Obesity		25.0		30.8		43.8		37.5	
Body Fat (%)	32.1±8.9		35.0±10.3		35.8±11.6		35.3±10.9		0.31
Lean Mass (kg)	31.2±8.5		42.1±10.0		37.5±10.4		35.4±7.6		0.001*
Fat Mass (kg)	16.2±10.2		25.1±15.8		24.1±16.4		21.7±13.0		0.01*
Diet, weight status, and adiposity at follow-up									
HEI total score	45.9±12.1		45.2±11.2		48.7±9.89		49.6±14.8		0.35
Kilocalories	1859.1±644.6		1759.5±683.0		1727.3±681.6		1749.7±644.9		0.78
Waist circumference (cm)	78.9±20.1		84.2±20.9		87.6±21.7		82.9±18.9		0.26
BMI Percentile	71.2±33.9		81.6±42.9		89.2±50.8		82.2±40.3		0.82
BMI category									0.58
Underweight		3.6		0.0		0.0		4.2	
Normal		55.4		56.4		40.6		50.0	
Overweight		16.0		12.8		12.5		8.3	
Obesity		25.0		30.8		46.9		37.5	
Body Fat (%)	31.1±8.9		35.6±10.5		36.8±12.2		34.2±11.4		0.06
Lean Mass (kg)	39.5±9.6		45.2±10.6		44.4±9.7		43.0±10.4		0.03*
Fat Mass (kg)	19.3±11.3		28.0±18.7		30.2±19.0		24.8±16.0		0.001*

*^Assessed using a One-Way analysis of variance (ANOVA) for continuous variables and a chi-squared or Fisher's exact test for categorical variables; TV=television; PD=portable device; HEI=Healthy Eating Index; BMI=Body Mass Index; *p<0.05*
